# Psychosocial Aspects During the First Wave of COVID-19 Infection in South Africa

**DOI:** 10.3389/fpsyt.2021.663758

**Published:** 2021-06-21

**Authors:** Antonio G. Lentoor, Mokoena Patronella Maepa

**Affiliations:** Department of Clinical Psychology, School of Medicine, Sefako Makgatho Health Sciences University, Pretoria, South Africa

**Keywords:** COVID-19, psychosocial, lockdown 2020, mental health, stress, threat, anxiety, depression

## Abstract

**Background:** In South Africa, as in many countries, the nationwide spread of COVID-19 caused a public health emergency that resulted in the government implementing necessary restrictive measures such as the nationwide lockdown as a way of containing the pandemic. Such restrictive measure, while necessary, can disrupt many aspects of people's lives resulting in unprecedented psychosocial distress.

**Aim:** The present study aims to describe the psychosocial health and situational factors associated with the novel coronavirus (COVID-19) in South Africa during the first wave of infection.

**Methods:** This cross-sectional survey, recruited a total of 203 participants through convenience sampling via online platforms—WhatsApp, Facebook, emails, etc.—during COVID-19 lockdown in the country. Through the snowball technique, participants from across South Africa completed the online survey that assessed socio-demographic information, risk perception, history of mental health, COVID-19-related stress, and fears during the lockdown (first wave).

**Results:** The majority of the participants who completed the survey were young, Black African, and female. Participants reported feelings of stress and anxiety (61.2%); stress about finances (39.5%); and feelings of sadness, anger, and/or frustration (31.6%) during the lockdown. Females compared to males were more likely to perceive COVID-19 as a risk to their household, $X_{\left(20\right)}^{2}$ = 45,844, *p* < 0.001 and community, $X_{\left(20\right)}^{2}$ = 40,047, *p* = 0.005. COVID-19 differentially impacted the mental health of participants with and without mental health diagnosis, $X_{\left(4\right)}^{2}$ = 16.596, *p* = 0.002. Participants with a prior mental health diagnosis reported significant extra stress during lockdown (*p* < 0.05).

**Conclusion:** The findings may be of significance to assist in the development of targeted psychosocial interventions to help people during and after the pandemic.

## Introduction

In January 2020, the World Health Organization (WHO) declared the outbreak of SARS-CoV-2, the virus that causes COVID-19, a public health emergency that raised an international concern (1). The disease has its origin in Wuhan, a Chinese city where it first appeared in December 2019 (2). By 2020, the international concern was heightened due to the rapid domestic and international spread of the virus. On January 30, 2020, the WHO declared the coronavirus a public health emergency of international concern (3). By end of February 2020, several countries had confirmed reported cases of the COVID-19 virus. On March 11, 2020, WHO declared the novel coronavirus disease a pandemic (4). Soon after, the Minister of Health of South Africa confirmed 13 positive cases of coronavirus as reported by the National Institute for Communicable Diseases, and by April 14, 2020, a total number of confirmed laboratory cases of COVID-19 of 2,272 with 27 reported COVID-19-related fatalities and 410 recoveries were reported (5).

It goes without saying that the first-ever global outbreak of the COVID-19 has negatively impacted the general population at large, with a considerable toll not only on healthcare needs but also various spheres of the life of individuals (6). A large proportion of the South African population has diverse and pre-existing vulnerable life situations, such as socioeconomic and health disparities. In line with the international response to contain the spread of COVID-19, South Africa went on a nationwide “hard” lockdown in March 2020, which inevitably singled crises and the general distress among the population. It is understood that a public health crisis of this nature cannot go without inflicting anxiety and fear throughout the population, resulting in a wide range of psychological health and social problems (7, 8). While lockdown is a necessity in situations such as this, it has caused distress, for example, the quarantine added stress, pay-cuts, unemployment, work and/or income uncertainty, fear, etc. Negative psychological impacts and unprecedented economic crises associated with the lockdown were reported in studies conducted outside of South Africa. For example, increased stress and anxiety related to COVID vulnerability, financial-related stress, and increased unemployment rates due to the loss of jobs after lockdown were reported in several countries (9). Previous findings showed that disruptions to people's work and lives were associated with negative effects on physical and mental health (10). While everyone is affected by the pandemic, poverty-ridden families, and communities are the hardest hit by the economic repercussions of the pandemic (11). Therefore, in this novel situation, social stress among individuals, families, and communities is unavoidable, and the risk for mental health problems is inevitable. The present study aims to describe the psychosocial health and situational factors associated with the novel coronavirus (COVID-19) in South Africa during the first wave of infection.

## Materials and Methods

### Research Design

In this prospective population-based cross-sectional study, individuals who were living in South Africa at the start of COVID-19, aged 18 years and older, and were able to provide informed consent, were eligible to participate in the study.

### Sample Size

The sample size was calculated with a 95% confidence level with a 5% margin of error and 50% response distribution, which was considered acceptable. A minimum of at least 300 people to complete the online survey was required. In terms of the numbers selected above, the sample size *n* and margin of error *E* are given by

$$\begin{array}{l} x=Z{\left({}^{c}{/}_{100}\right)}^{2}r(100-r)n{=}^{Nx}{/}_{\left[(\text{N}-1){\text{E}}^{2}+\text{x}\right]}\\ \;E=Sqrt{[}^{(\text{N-n})\text{x}}{/}_{\text{n}(\text{N}-1)}], \end{array}$$

where *N* is the population size, *r* is the fraction of responses that you are interested in, and *Z*(*c*/100) is the critical value for the confidence level *c*.

### Study Measures

The online survey consisting of questions that asked about socio-demographic, psychological, and health status prior to and during COVID-19 were constructed.

#### Sociodemographics

The participants' age group, gender, ethnicity, level of education, marital status, province of living, and employment status were collected.

#### Mental Health

Participants were asked whether they had ever been diagnosed with a mental health problem such as depression and anxiety (*Yes, No*), whether they are currently being treated for a mental health problem (*Yes, No*), and what current treatment they are receiving (*Psychiatric medication only, Psychotherapy only, Combined psychiatric medication and psychotherapy, Other*).

#### COVID-19-Related Stress

Participants were asked whether they were struggling with lockdown [*feelings of stress and anxious (anxiety), sadness, anger, and/or frustration (depressed); problems in family relationships; problems in romantic relationships; stress about finances; thoughts and/or feelings of suicide; or increased use of alcohol/cigarettes/drugs*].

#### Seeking Mental Healthcare During the Lockdown

Participants were asked whether they sought mental healthcare for COVID-19-lockdown-related difficulties (*Yes, No*).

#### COVID-19 Threat and Fear

Participants were assessed how much a threat COVID-19 was to their country, city, community, and household (*Yes, No*); if they were afraid of contracting COVID-19 (*Yes, No*); if they worried for their family or friends contracting COVID-19 (*Yes, No*).

### Procedure and Data Collection

In line with the South African government's recommendation of social distancing and national lockdown to minimize face-to-face contact, the use of the electronic platform, an online survey was deemed the most feasible method for the collection of data. All participants were invited to complete an online survey that was distributed on various social media platforms, including Facebook, Twitter, university websites, WhatsApp, forums, etc. Using a snowball sampling strategy, an anonymous online questionnaire was distributed for data collection during the period of March 5, 2020 to November 31, 2020, the first wave of infection.

### Ethical Consideration

Ethical approval was received from Sefako Makgatho University Research Ethics Committee (SMUREC/M/73/2020: IR), and the research was carried out in accordance with the Declaration of Helsinki of the World Medical Association, and participants gave informed written consent.

### Statistical Analysis

Descriptive statistics were used to compile socio-demographic and health profiles of the study sample and were expressed in mean (*M*), standard deviations (*SD*), and frequency data. Inferential statistics such chi-square test (nominal data) for differences in socio-demographic, psychosocial, and health factors were conducted in the study. All analyses were conducted using IBM SPSS Statistics version 20 (IBM Corporation, Armonk, NY, USA.), with a level of significance set at 0.05, two-tailed.

## Results

A total of 306 people age 18 and older consented and completed the online survey. Basic socio-demographic characteristics were presented in Table 1. Of the participants, 45.8% were Black African, 15.4% White, 6.9% Indian/Asian, and 3.3% Colored. The majority of the participants who completed the survey were female (71.2%) compared to 28% male and were married (49%). The majority of the participants who completed the online survey lived in Gauteng (48.4%) followed by Limpopo province (12.7%) during the lockdown of the first wave of the pandemic.

**Table 1:** Sociodemographic characteristics of all the participants in the study (*N* = 306).

**Characteristics**	**All participants**		**Female**		**Male**	
	***n***	***(%)***	***n***	***(%)***	***n***	***(%)***
**Age (years)**						
18–29	84	27.5	71	23.2	13	4.2
30–44	138	45.1	90	29.4	48	15.7
45–54	47	15.4	33	10.8	14	4.6
55–64	29	9.5	20	6,5	9	2.9
65+	8	2.5	4	1.3	4	1.3
**Race/Ethnicity**						
Black African	140	45.8	96	31.4	44	14.4
White	47	15.4	37	12.1	10	3.3
Colored	10	3,3	8	2.6	2	0,7
Indian/Asian	21	6.9	13	4.2	8	2.6
**Relationship status**						
Dating	66	21.6	36	11.8	30	9.8
Married	150	49.0	111	36.3	39	12.7
Divorced	10	3.3	9	2.9	1	0.3
Single	100	32.7	77	25.2	23	7.5
**Highest level of education**						
High school	22	7.2	14	4.6	8	2.6
Bachelor's degree	86	28.1	58	19.0	28	9.2
Post-graduate degree	197	64.4	146	47.7	51	16.7
**Occupation status**						
Employed	226	73.9	160	52.3	66	21.6
Unemployed	27	8.8	21	6.9	6	2.0
Other (student)	45	14.7	35	11.4	10	3.3
**Province**						
Eastern Cape	18	5.9	12	3.9	6	2.0
Free State	7	2.3	4	1.3	3	1.0
Gauteng	148	48.4	106	34.6	42	13.7
KwaZulu Natal	26	8.5	21	6.9	5	1.6
Limpopo	39	12.7	31	10.1	8	2.6
Mpumalanga	11	3.6	5	1.6	6	2.0
North West	17	5.6	9	2.9	8	2.6
Northern Cape	4	1.3	2	0.7	2	0.7
Western Cape	35	11.4	28	9.2	7	2.3
**Required to work in lockdown**						
Yes	215	70.3	149	48.7	66	21.6
No	70	22.9	53	17.3	17	5.6
**History of mental illness**						
Diagnosed with mental illness	53	16.1	40	17.2	11	11.9

Of all the participants, 61.2% reported feelings of stress and anxiety, while 39.5% reported stress about finances, and 31.6% reported feelings of sadness, anger, and/or frustration since the beginning and/or during the lockdown. The percentage of all three indicators was comparatively higher among women than men. In comparison, the higher age group (participants 45 years and older) reported higher percentages of stress, anxiety, and depressive symptoms than the younger age group (participants 18–44 years), but the differences were not statistically significant (*p* = 0.666).

**Figure 1:**
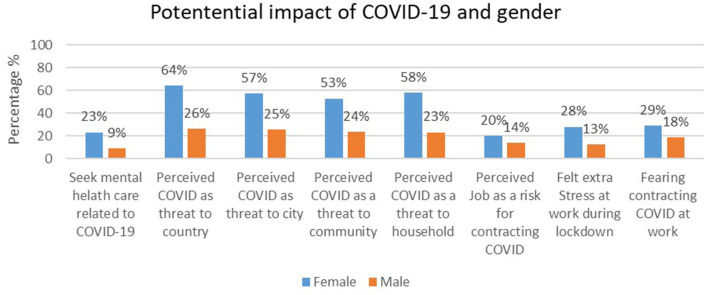
Impact of COVID-19 by gender.

Of all the participants, only 32% reported seeking out mental healthcare for COVID-19-related challenges during the lockdown (Figure 1). A significantly higher percentage of females compared to males were seeking out mental healthcare specifically related to challenges experienced during COVID-19 lockdown (23 vs. 9%, *p* < 0.001) (Table 2). The majority of the participants considered COVID-19 a serious threat either to communities (76.6%) and households (80.9%). Females compared to males were more likely to perceive COVID-19 as a risk to their household, $X_{\left(20\right)}^{2}$ = 45,844, *p* < 0.001 and community, $X_{\left(20\right)}^{2}$ = 40,047, *p* = 0.005.

**Table 2:** Comparison of COVID-19-related impact by gender and age.

	**Gender**			**Age**		
**Variables**	***X*^**2**^**	***df***	***p***	***X*^**2**^**	***df***	***P***
Continue work during COVID-19	45.96	8	0.000^***^			
Job putting me at risk for COVID-19	24.98	8	0.002^**^	5.93	2	0.051^*^
Extra stress and anxiety at work	15.40	8	0.052^*^			
Fear and worry contracting COVID-19 at work	26.00	8	0.001^***^	10.91	2	0.004^**^
Perceived threat to household	45.84	20	0.000^***^			
Perceived threat to community	40.04	20	0.005^**^			
Seek COVID-related mental healthcare	50.01	8	0.000^***^			
Previous mental health problem	259.13	8	0.000^***^			
Treated for mental health problem	48.73	8	0.000^***^			

*X^2^, chi-square; df, degrees of freedom*.

*Levels of significance*:

* *p < 0.05*,

** *p < 0.01*,

*** *p < 0.001*.

Of the participants who completed the survey, over 70% were still required to continue working during the lockdown. Moreover, over 60% reported their work to be stressful, while over 40% reported extra stress at work during the COVID-19, and close to 34% believed that their work was increasing their vulnerability for COVID-19. Fear of contracting COVID-19 through the job (or work) was reported by 47.5% of the participants (Figure 1), but they felt that they had little control over it.

Of the overall sample, a total of 53 (16.1%) of the participants had a mental health condition that was diagnosed prior to COVID-19, and 10.4% reported receiving psychiatric care. Psychotropic medication only (5.5%), psychotherapy only (4.8%), or combined psychiatric and psychotherapy (0.9%) were the commonest modalities of treatment reported by the participants. Compared with males, females were more likely to report a history of mental illness $X_{\left(8\right)}^{2}$ = 40.882, *p* < 0.001, with depression and anxiety being the more commonly diagnosed disorders. COVID-19 had a differential impact on the mental health of participants with and without mental health diagnoses, $X_{\left(4\right)}^{2}$ = 16.596, *p* = 0.002. Participants with a pre-existing mental health diagnosis were more likely to report extra stress during COVID-19 (*p* = 0.002), consider work stressful (*p* < 0.001) and seek out mental healthcare during the lockdown (*p* < 0.001) (Table 3).

**Table 3:** Relationship between previous diagnosed mental health problem and impact of COVID-19.

**Variable**	**Diagnosed mental illness**		
	***X*^**2**^**	***df***	***p***
Job putting me at risk for COVID-19	5.596	4	0.232
Extra stress and anxiety at work	17.096	4	0.002^**^
Fear and worry contracting COVID-19 at work	6.926	4	0.140
Perceived threat to household	33.480	10	0.000^***^
Perceived threat to community	36.372	10	0.000^***^
Seek COVID-related mental healthcare	22.542	10	0.000^***^

*X^2^, chi-square; df, degrees of freedom*.

*Levels of significance*:

** *p < 0.01*,

*** *p < 0.001*.

## Discussion

This study confirms a large percentage of participants who completed the study survey experienced adverse psychosocial effects associated with COVID-19 and lockdown in a sample of South Africans. This finding is similar to studies conducted in several countries (2, 12–15). Notably, our study found that more than a third of the participants reported psychological problems as a result of the pandemic. In this study, stress and anxiety; stress about finances; and depressive symptoms were significantly higher in women than in men. Similar findings were observed elsewhere (16). Hou et al. (17), in an online recruited study of 3,088 participants from across China, found that females compared to males reported more and severe stress and anxiety during the COVID-19 epidemic. Similar gender differences were observed in the findings by Wang and Tang in their cross-sectional survey of 4,788 men and women from eight provinces across China (18). The researchers found that women comparatively to men reported higher percentages of hopelessness, loneliness, and depression. It is worth noting that while these findings may suggest a gender difference in the mental health vulnerability associated with the pandemic, further research is required to clearly unpack this. Notwithstanding, it is well-established that women are at greater risk for mental health problems than men, especially for mood and anxiety disorders (19, 20). In the context of the pandemic, women are at an increased risk of poor mental health outcomes due to vulnerability of intimate partner violence, income insecurity, and the likelihood of loss of livelihood associated with the COVID-19 lockdown (21). Although we did not collect data on the presence of children, it may be the case that some female participants reported higher levels of stress than male participants because the lockdown exacerbated the gender division of family work (i.e., caring of children, chores, etc.) within the households. With the lockdown and the closures of daycares and businesses, it meant women may be doing family work that would have previously been outsourced or taking on more childcare responsibilities, and more homeschooling than men, while outside sources of help such as cleaners, tutors, and support from extended family are not available. The finding suggests that women may be more stressed during the lockdown because they may be over-proportionately burdened by additional responsibilities in the home.

Similar to Nwachukwu et al. our study found that participants in the lower age groups (18–29 years) reported relatively higher percentages of stress, anxiety, and depressive symptomatology (16). However, a significant age effect was only observed for fear and anxiety about contractingCOVID-19 at work and perceiving one's job as a risk for contracting COVID-19. Importantly in contrast to Nwachukwu and colleagues, the participants in our study were spread over a wider younger age 18–39 years, with a younger average (16). Our study, similar to other studies (16, 18, 22) had a lower representation of the elderly population, and this underrepresentation may be a potential bias in this study that limits generalizability for those older than 55 years of age. Another possible explanation is that persons in the younger age group may have perceived their academic, social, occupational, and economic prospects to be more threatened by COVID-19 compared to older individuals (23).

This study also supports the finding that more women are seeking mental health support as the pandemic pushes up stress, anxiety, and depression (24).

Our study finding is consistent with studies elsewhere that suggest the spread of coronavirus is perceived as a threat and a risk (25, 26). Similar to Kanovsky and Halamová (25), our study found that participants who go out to work reported a higher percentage of perceived likelihood of contracting COVID-19. Regardless of actual exposure, fear of infection and the risk of transmitting infection between work and family was a major concern for the majority of the participant, a finding that is aligned with previous studies (27, 28). Interestingly, in a study, Niño et al. (29) found that race and ethnicity, gender, and age play a significant role in the threat and fear perceptions of COVID-19. Remaining to be unpacked in future studies is the underlying factors that can explain differences in perception of risk during an infectious disease outbreak.

Consistent with previous studies, our finding confirms the potential impact of the COVID-19 pandemic on participants with a self-reported history of mental health problems (28, 30).

In light of the above findings, it is imperative for the government to prioritize the mental health needs of its people by providing the necessary psychosocial health support. The prolonged lockdown situation may continue to contribute to increasing levels of distress, fear of losing jobs, and stress associated with restricted movement outside of the home and increasing responsibility within the home context. Particular attention should be given to multiple role handling that is particularly relevant for women who continue to engage in more child care, homeschooling of children, and domestic responsibilities, while also performing full-time jobs from home. Psychologists and other mental health practitioners can play an important role in meeting the mental health needs of the general population during the ongoing pandemic by assisting governments in tailoring psychosocial interventions. Online or smartphone–based psychological interventions have been promoted as an effective solution in this pressing time of global emergency. We, therefore, recommend the active and ongoing participation of government, mental health policymakers, and mental health professionals as a joint effort during this critical time to ensure the psychosocial well-being of all people (31). Employers can also directly support employees, especially providing women with flexible working schedules, pay women equal wages and spare them the stress, and make mental healthcare accessible through employer-based healthcare plans.

### Limitations of the Study

Our study is not without limitations. First, the issue of generalizability is highlighted in the small sample size that is not representative of the South African population. Due to the nationwide lockdown and movement restriction, we had to rely solely on collecting the data *via* an online survey, which might not represent the population as uniformly as one would like in such studies. This may have introduced several issues such as the inability to reach the wider and perhaps the most marginalized population (i.e., the poor, elderly, disabled, and people with limited or no education). Also, those individuals who are not computer literate, who do not have access to the internet, smartphones, or relevant digital resources were also limited from participating. Second, the data collected for this study is limited only to the first wave of infection in South Africa. Furthermore, data were self-reported, and thus the potential of reporting bias may have been introduced. In addition, the study reported on a limited selection of symptoms. Future studies can address this by incorporating a broad range of psychosocial consequences of COVID-19. Third, we did not ask if the participants or their families had exposure to COVID-19. Finally, this study was based on cross-sectional data, and the associations cannot indicate causality. Despite these limitations, the findings of this study contribute to our understanding of the psychosocial consequences of COVID-19.

## Conclusion

A large percentage of the participants had psychosocial problems associated with the pandemic lockdown. Women as opposed to men and patients with a prior mental health diagnosis are especially vulnerable to the adverse outcomes of COVID-19. In conclusion, future research should explore the psychosocial health changes of a cohort over time as the pandemic evolves in South Africa.

## Data Availability Statement

The raw data supporting the conclusions of this article will be made available by the authors, without undue reservation.

## Ethics Statement

The studies involving human participants were reviewed and approved by Sefako Makgatho University Research Ethics Committee (SMUREC/M/73/2020: IR). The patients/participants provided their written informed consent to participate in this study.

## Author Contributions

AL and MM contributed to conception and design of the study and wrote sections of manuscripts. AL performed statistical analysis and wrote the first draft of the manuscripts. Both authors contributed to manuscript revision, read, and approved the submitted version.

## Conflict of Interest

The authors declare that the research was conducted in the absence of any commercial or financial relationships that could be construed as a potential conflict of interest.
